# Delayed slipped capital femoral epiphysis with hypothyroidism in children: A case report

**DOI:** 10.1097/MD.0000000000041789

**Published:** 2025-03-07

**Authors:** Qinggang Zhao, Xingxi Hu, Yihao Lei, Fengyong Guo, Kaiyu Hou, Yongcheng Deng, Zhong Chen, Junliang Jiang, Ge Chen

**Affiliations:** a Department of Orthopedics and Traumatology, The Affiliated Hospital of Yunnan University, Kunming, Yunnan Province, China.

**Keywords:** endocrine disorders, hip pain, pediatric orthopedics, slipped capital femoral epiphysis, surgical intervention

## Abstract

**Rationale:**

Slipped capital femoral epiphysis (SCFE) is a common adolescent hip disorder, but its association with hypothyroidism remains rare and poorly understood. This case highlights the importance of considering endocrine disorders, such as hypothyroidism, as potential risk factors in atypical SCFE presentations, particularly when trauma or other common etiologies are absent.

**Patient concerns:**

A 14-year-old boy presented with progressive, nontraumatic left hip pain that worsened over 6 weeks, eventually leading to complete immobility. Initially managed unsuccessfully with traditional Chinese medicine, the patient sought care at a trauma center before referral to our hospital.

**Diagnoses:**

Imaging confirmed a delayed, moderate SCFE of the left hip, characterized by posteromedial displacement of the femoral epiphysis. Laboratory tests revealed significant hypothyroidism, with elevated thyroid-stimulating hormone (>100 μIU/mL) and reduced thyroid hormone levels (FT4, FT3, TT3, TT4).

**Interventions:**

The patient underwent open reduction and internal fixation using 4 Kirschner wires via an anterolateral approach, followed by immobilization in a hip spica cast for 2 months. Postoperatively, hypothyroidism was managed with levothyroxine (50 μg/day) under endocrinological supervision.

**Outcomes:**

After 2 years of follow-up, the patient achieved full functional recovery with no evidence of complications, such as avascular necrosis or residual deformity. Bone union was complete by 8 weeks, and normal activity resumed within 5.5 months.

**Lessons:**

This case underscores the need to screen for thyroid dysfunction in pediatric SCFE patients lacking typical risk factors, such as trauma or obesity. Early recognition and interdisciplinary management of both orthopedic and endocrine aspects can optimize outcomes and prevent long-term complications, emphasizing the value of a comprehensive diagnostic approach.

## 1. Introduction

Slipped capital femoral epiphysis (SCFE) is a developmental orthopedic condition with an incidence rate of 0.2 per 100,000 children, characterized by a male-to-female ratio of approximately 1.5:1.^[1]^ The etiology of SCFE remains a subject of ongoing debate, particularly regarding its association with endocrine disorders such as hypothyroidism. While earlier studies suggested no correlation between SCFE and hypothyroidism,^[2]^ more recent case reports have identified a potential link between the 2 conditions.^[3,4]^ Notably, Pavone et al^[5]^ emphasized the importance of timely diagnosis and management to avoid complications in cases of SCFE, particularly in patients with underlying endocrine disorders such as hypothyroidism.

This report presents an exceptional case of delayed SCFE in a 14-year-old male patient with hypothyroidism. Following the diagnosis, the patient underwent open reduction with internal fixation using 4 Kirschner wires, followed by immobilization with a hip spica cast for 2 months. After 2 years of follow-up, the patient demonstrated satisfactory treatment outcomes.

## 2. Case presentation

A 14-year-old Chinese boy suddenly experienced dull pain in his left hip while running at school. About 1 week later, the pain recurred and did not subside with rest. During this period, the patient was still able to walk independently. After 3 weeks, despite the pain intensified and affected walking, the patient did not inform his parents of the problem. Three weeks later, his father took him to a local Chinese medicine clinic, where the doctor tried to take a “cupping” to relieve the pain in his left hip, but this treatment was unsuccessful. Six weeks after the initial onset of pain, the patient was completely unable to walk. The patient was then brought to a local trauma center and diagnosed with a “fracture in the epiphysis of the left femoral head.” The local doctors did not suspect any underlying endocrine issues. Due to the high risk of surgery, he visited our hospital with his father half a month later.

**Figure 1.**
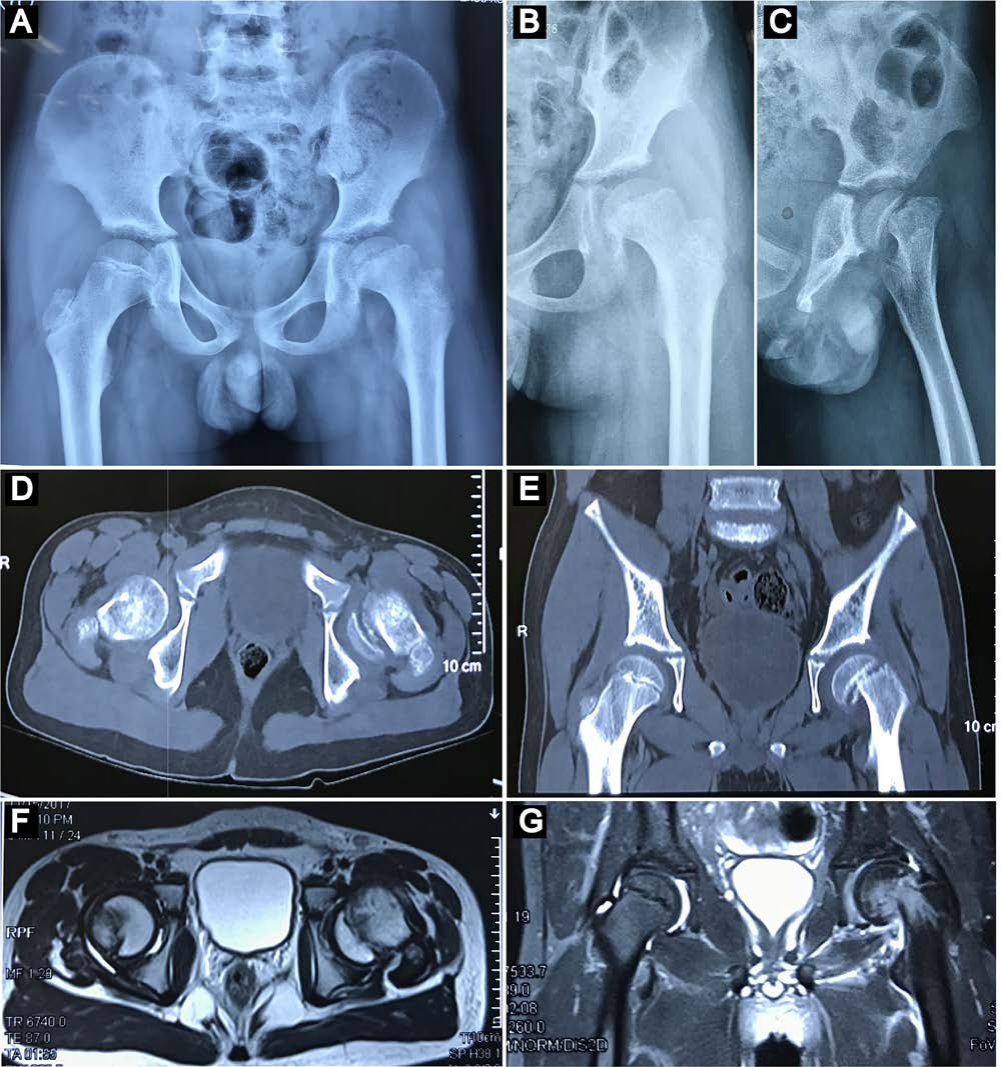
Imaging data at admission. (A) Antero-posterior pelvic radiograph. (B) Antero-posterior radiograph of the left hip. (C) Lateral radiograph of the left hip. (D) Axial plane of CT scan. (E) Coronal plane of CT scan. (F) Axial plane of MRI. (G) Coronal plane of MRI. MRI = magnetic resonance imaging.

The patient arrived at a height of 143 cm and weight of 35 kg (body mass index = 17.11). He had no history of medication use, trauma, seizures, steatorrhea, kidney diseases, bone metabolic disorders, nor endocrine diseases. Both the blood test results and erythrocyte sedimentation rate were normal. The patient maintained a regular lifestyle and routine diet, with no abnormal family history. Physical examination revealed limited range of motion in the left hip, including flexion, adduction, internal rotation, and abduction. Plain radiographs and CT scans demonstrated a delayed, moderately slipped capital femoral epiphysis in the left hip (Fig. 1A–E). Magnetic resonance imaging (MRI) showed callus formation in the epiphyseal area, without evidence of femoral head necrosis (Fig. 1F, G). These radiographic evaluations were conducted and interpreted by a radiologist, who was responsible for the initial assessment and measurement of the radiographic parameters. The same evaluator was involved in all subsequent radiographic evaluations.

The blood test results were as follow: serum vitamin D levels were 35.36 ng/mL (60 > normal range > 11 ng/mL); serum levels of thyroid stimulating hormone > 100 μIU/mL (0.35–5.5 μIU/mL; the free thyroxine (FT4) = 3.64 pmol/L (22 pmol/L > normal range > 10 pmol/L); free triiodothyronine = 1.77 pmol/L (7.0 pmol/L > normal being > 3.50 pmol/L); total triiodothyronine = 0.33 ng/dL (1.9 ng/dL > normal being > 0.8 ng/dL); total thyroxine < 0.50 ng/dL (12 ng/dL > normal range > 4.5 ng/dL). Accordingly, the patient was diagnosed with delayed moderately slipped capital femoral epiphysis of the left hip associated with hypothyroidism, and surgery was deemed necessary. The treatment was managed by a pediatric orthopedic surgeon, who carefully considered the patient’s age, the severity of the epiphyseal displacement, and the potential risk of complications such as avascular necrosis.

**Figure 2.**
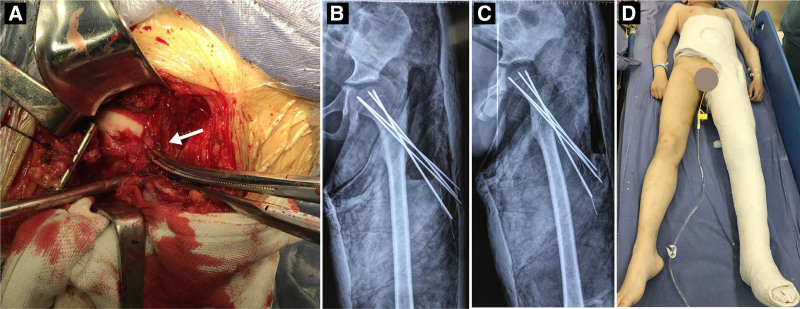
Imaging data during operation. (A) Open reduction and internal fixation using an anterolateral approach. The white arrow indicated the slipped capital femoral epiphysis. (B) Antero-posterior radiograph of the left hip. (C) Lateral radiograph of the left hip. (D) One-and-a-half hip spica cast.

Considering the significant posteromedial displacement of the epiphysis, open reduction and internal fixation were performed using the anterolateral approach. A T-shaped incision was made to open the joint capsule and a small amount of extravasated blood was observed. The left femoral head was dislocated downward and backward and surrounded by a few calluses (Fig. 2A). Partial cartilage of the femoral head was worn. The calluses around the epiphysis were cleaned and the femoral head was reduced. Subsequently, four 2.0-mm threaded K-wires were percutaneously inserted to fix the femoral head under C-arm fluoroscopy (Fig. 2B, C). The threaded K-wires provided enhanced stability for the femoral head. The tail of the K-wires was retained on the skin surface, after which the surgical incision was closed. Intraoperative blood loss was approximately 400 mL. To further stabilize the internal fixation, a one-and-a-half hip spica cast was applied (Fig. 2D).

**Figure 3.**
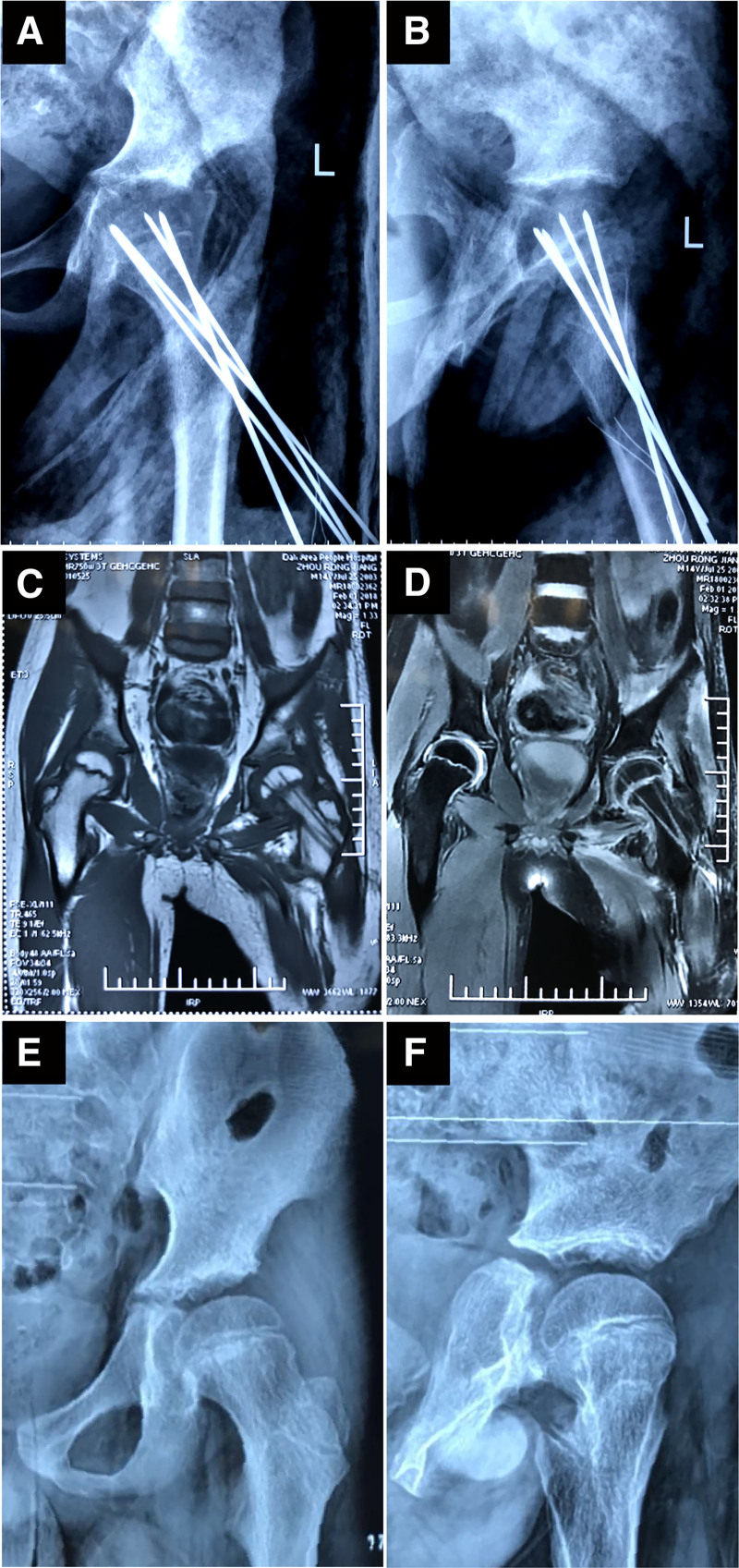
Imaging data during follow-up. (A) Antero-posterior radiograph of the left hip at 8 weeks postoperative. (B) Lateral radiograph of the left hip at 8 weeks postoperative. (C) Axial plane of MRI at 3 months postoperative. (D) Coronal plane of MRI at 3 months postoperative. (E) Antero-posterior radiograph of the left hip at 3 months postoperative. (F) Lateral radiograph of the left hip at 3 months postoperative. MRI = magnetic resonance imaging.

The patient began taking levothyroxine sodium tablets (50 μg/day) on the first postoperative day (recommended by the endocrinologist). Signs of bone union were evident at 4 weeks, and complete union was achieved at 8 weeks (Fig. 3A, B), while all the K-wires and cast were removed. MRI showed no abnormalities in bone morphology or joints (Fig. 3C, D). The patient completely resumed normal activity 5 and a half months later. After 2 years of follow-up, the patient did not develop avascular necrosis of the femoral head or residual limb deformity (Fig. 3E, F).

## 3. Discussion

Albeit a rare disease around 20% to 80% of patients present with bilateral SCFE. The second SCFE usually occurs 1 year after the first slip.^[6]^ Children diagnosed with unilateral SCFE have a 2335 times greater risk of developing a subsequent contralateral slip than average children.^[7]^ Approximately 40% of patients with unilateral SCFE exhibit radiographic features of an undiagnosed contralateral slip.^[8]^

From the researchers’ perspective, the reason for this predicament is that SCFE is not only a characterization of fracture in children but may also be a pathological manifestation of the skeletal system secondary to the patient’s obesity, vitamin D deficiency, and growth hormone deficiency. Many orthopedic surgeons currently concentrate on the management of SCFE alone but ignore the metabolic disorders causing SCFE, which could result in a resurgence of SCFE and indicate the occurrence in the contralateral hip, as well as for shadow serious secondary joint diseases in adulthood (e.g., necrosis of the femoral head and osteoarthritis). Interestingly, neither obedience nor vitamin D deficiency was observed in this patient. Regarding the positive correlation between vitamin D deficiency and thyroiditis, hypothyroidism, and thyroid immune diseases,^[9]^ the patient’s SCFE was thought to be associated with hypothyroidism. Blood tests verified this hypothesis.

The relationship between SCFE and hypothyroidism has not been extensively validated in the literature.^[2–4]^ This case of delayed SCFE associated with hypothyroidism adds to the limited body of evidence that supports this correlation. It is critical to recognize the increased incidence of develop avascular necrosis in children with SCFE, particularly in delayed cases, which pose an even greater risk.^[6]^ For this reason, we opted for open reduction using 4 Kirschner wires, followed by immobilization with a hip spica cast for 2 months. This dual immobilization strategy ensures an adequate blood supply to the epiphysis and femoral head, contributing to favorable clinical outcomes.

The management of this case demonstrated several strengths, including a comprehensive approach that utilized open reduction and internal fixation with Kirschner wires, effectively addressing the delayed SCFE and ensuring stability and adequate blood supply to the femoral head. Additionally, the consideration of hypothyroidism as a contributing factor allowed for a tailored management plan, potentially preventing future complications. However, the case also has limitations; the correlation between SCFE and hypothyroidism is not widely validated in the literature, and the findings are based on a single case, which restricts generalizability. Furthermore, the absence of common risk factors, such as vitamin D deficiency, may introduce bias in interpreting the relationship between SCFE and endocrine disorders. Lastly, long-term follow-up data are necessary to assess the treatment’s effectiveness and monitor for potential complications.

This case report is limited by its single-center design and the relatively small sample size, which restricts the generalizability of the findings. The association between hypothyroidism and SCFE, as well as the outcomes of treatment with K-wires, need to be confirmed in larger, multi-center, prospective studies. Future research with broader patient cohorts will be essential to validate these findings and determine whether the observed relationship holds true across different populations. Additionally, longer-term studies are needed to evaluate the sustained effectiveness and safety of the treatment approach employed in this case, including the use of threaded K-wires for internal fixation.

To the best of our knowledge, this case represents a rare instance of delayed SCFE in a child with hypothyroidism without any preceding trauma. In conclusion, pediatric patients presenting with hip, thigh, or knee pain without obvious trauma should be evaluated for potential underlying endocrine disorders, particularly if they have a history of thyroid dysfunction, obesity, or vitamin D deficiency. If no apparent trauma is identified, it is essential to investigate both the vitamin D levels and thyroid function. In cases of delayed SCFE, open reduction and internal fixation using Kirschner wires combined with hip plaster fixation may be an effective treatment option.

## Author contributions

**Conceptualization:** Xingxi Hu.

**Formal analysis:** Ge Chen.

**Investigation:** Fengyong Guo, Yihao Lei.

**Methodology:** Xingxi Hu.

**Resources:** Junliang Jiang.

**Writing – original draft:** Qinggang Zhao.

**Writing – review & editing:** Kaiyu Hou, Yongcheng Deng, Zhong Chen.
